# SciComm Optimizer for Policy Engagement: a randomized controlled trial of the SCOPE model on state legislators’ research use in public discourse

**DOI:** 10.1186/s13012-023-01268-1

**Published:** 2023-05-05

**Authors:** J. Taylor Scott, K. Megan Collier, Jessica Pugel, Patrick O’Neill, Elizabeth C. Long, Mary A. Fernandes, Katherine Cruz, Brittany Gay, Cagla Giray, D. Max Crowley

**Affiliations:** 1 Evidence-to-Impact Collaborative, The Pennsylvania State University, State College, USA; 2 Social Work Department, Boston College, Chestnut Hill, USA; 3 Psychology Department, Teachers College at Columbia University, New York City, USA; 4 Department of Psychology, Georgia State University, Atlanta, USA; 5 Department of Health Policy and Management, Johns Hopkins Bloomberg School of Public Health, Baltimore, USA; 6 Center for Health Security, John Hopkins Bloomberg School of Public Health, Baltimore, USA

**Keywords:** Dissemination, Use of research evidence, Policymaking, Research translation, Science communication

## Abstract

**Background:**

While prior work has revealed conditions that foster policymakers’ use of research evidence, few studies have rigorously investigated the effectiveness of theory-based practices. Specifically, policymakers are most apt to use research evidence when it is timely, relevant, brief, and messaged appropriately, as well as when it facilitates interactive engagement. This study sought to experimentally evaluate an enhanced research dissemination intervention, known as the SciComm Optimizer for Policy Engagement (SCOPE), implemented during the COVID-19 pandemic among US state legislators.

**Methods:**

State legislators assigned to health committees and their staff were randomized to receive the SCOPE intervention. This involved providing academic researchers with a pathway for translating and disseminating research relevant to current legislative priorities via fact sheets emailed directly to officials. The intervention occurred April 2020–March 2021. Research language was measured in state legislators’ social media posts.

**Results:**

Legislators randomized to receive the intervention, relative to the control group, produced 24% more social media posts containing research language related to COVID-19. Secondary analyses revealed that these findings were driven by two different types of research language. Intervention officials produced 67% more COVID-related social media posts referencing technical language (e.g., statistical methods), as well as 28% more posts that referenced research-based concepts. However, they produced 31% fewer posts that referenced creating or disseminating new knowledge.

**Conclusions:**

This study suggests that strategic, targeted science communication efforts may have the potential to change state legislators’ public discourse and use of evidence. Strategic science communication efforts are particularly needed in light of the role government officials have played in communicating about the pandemic to the general public.

**Supplementary Information:**

The online version contains supplementary material available at 10.1186/s13012-023-01268-1.

Contributions to the literature

- Very little previous work has experimentally tested the impact of research dissemination activities on policymakers’ use of research evidence, and this study illustrates a feasible method of doing so
- This study builds on descriptive research that has shed light on tactics to improve research translation for policymakers by designing and testing an “enhanced dissemination” that facilitates direct researchers and state legislators via email.
- This study demonstrates that a theory-based model, the SciComm Optimizer for Policy Engagement (SCOPE), was effective in increasing state legislators’ use of research language in social media posts related to COVID-19.

## Background

The COVID-19 pandemic has laid bare the costs of failing to access and use scientific research that informs governmental action and communication [1–3]. This has led to widespread calls to evaluate effective science communication and dissemination interventions [4, 5]. Government officials have played a particularly meaningful role in communicating about the pandemic via social media, which has been critical for disseminating accurate information but has, in some cases, contributed to the spread of misinformation [4]. Despite existing strategic models of science communication, much of the previous work in this area has been retrospective and relied on self-report of how policymakers access and use research. Few experimental studies have investigated effective strategies to support policymakers’ use of research evidence (URE). This study seeks to prospectively and experimentally evaluate an enhanced science communication intervention to support US legislators’ use of public health research—to our knowledge, the first of its kind [6]. Specifically, we investigate legislators’ URE via social media, given its role in both combatting and perpetuating misinformation during the pandemic.

### Study context: the COVID-19 pandemic, misinformation, and politics

In 2020, the world was gripped by the COVID-19 pandemic, which not only disrupted the normal social order (e.g., “work from home”), but also altered the public’s degree of exposure to and engagement with scientific research. The intense need for supporting the rapid uptake of high-quality research evidence encouraged responses from the scientific community. Even before the pandemic, there were significant concerns among the scientific community that a “war on science” was politicizing and delegitimizing public perception of scientific credibility [7]. Despite this popular narrative [8, 9], US and international public opinion of science remained positive among the general public [10, 11] and the policy community [12], plausibly because the “war” was spurred by the beliefs of vocal individuals concerned about vaccines and other specific issues [10]. Immediately in the wake of the pandemic, public trust in science fluctuated [2] and declined over time, especially among Republicans [13]. Despite these recent trends, substantial research has shown that most policymakers across the aisle have reported that research is valuable to their work, and are eager to use research in their work even if approaching it in different ways [14–17]. Lawmakers also report difficulty accessing unbiased, nonpartisan, or agenda-neutral scientific evidence [18, 19] and view university-based research as more trustworthy than that from advocacy groups and think tanks [20]. This is evidenced in studies with state legislators, including one analysis indicating over half of state legislators as highly valuing research evidence, but less than a quarter reporting access to high-quality research evidence [12].

Mutual mistrust and a lack of scaffolded opportunities to facilitate interactions between academic researchers and policymakers are prominent barriers to URE [21, 22]. It is critical that public health practitioners recognize that trust in science is built through interpersonal relationships. Misunderstanding the need for relational approaches to research translation have inspired simplistic, one-way dissemination efforts that “push” information from research organizations without regard to policymakers’ current needs [23]. Such impersonal science communication approaches are ultimately inconsistent with best practice—which should involve collaborative and interactive approaches for tailoring responses to policymakers’ goals and evidence needs [21]. In particular, research that is timely and relevant is most likely to be used by policymakers [24].

### Prior dissemination research

A meaningful body of research has shed light on conditions that foster URE in the policymaking process [21, 25–27], yet few studies have experimentally investigated best practices for disseminating research evidence among policymakers, and virtually none have examined the impact of research dissemination on policymakers’ public discourse. Meaningful experimental work has been done recently to improve the way that research is communicated to policymakers. For instance, evoking emotion and threat-based language [28], state-tailored economic evidence [29], and cueing relevance to the state or legislator [24] have been shown to increase legislators’ access of research evidence via email. However, it is unclear what measurable impact such disseminations could have on policymaker URE.

Previous experimental studies with legislative subjects have investigated from whom policymakers learn and how access to information can influence their behavior [23]. For instance, a recent study found that participation in a bipartisan group wherein policymakers discussed their proposed bills with one another increased support for those bills [30]. A subsequent research dissemination study found that when provided with a non-partisan technical briefing on a bill, legislators were 60% more likely to support that bill [31]. Such studies provide evidence that communication can affect policymakers’ behavior [23, 30, 31]. We seek to build on this literature base by further shedding light on how disseminated research information may affect legislators’ URE in their public discourse, specifically in their social media posts.

### Conceptual framework

This work draws upon multiple theoretical frameworks for understanding how, why, and when policymakers use research, as well as best practices for disseminating research evidence as revealed in extant literature. Foremost, corresponding with Brownson’s Model of Dissemination Research [32], the current study *source* involves researchers who send *messages* about research synthesis in fact sheets, using emails as the *channel* to deliver the message to an *audience* of US state legislators and their staff. Embedded in this investigation is investigators’ recognition of a widely used typology describing *how* policymakers use evidence [30, 31], which includes evidence that directly informs policy development (i.e., instrumental use) or how policymakers think about causes and consequences of problems (i.e., conceptual use). Policies may also leverage research or evaluation methods (i.e., process use) or research may be used to justify preconceived policy stances (i.e., tactical use [19, 20]).

John Kingdon’s Three Streams Model is fundamental to understanding *when* research evidence is deemed timely based on socio-political factors [35]. Timeliness and relevance of research correspond with these opportunity windows, which has been emphasized for policymakers’ URE [36, 37]. To do this, science dissemination efforts must focus on end-users’ needs, which is exemplified by an array of practices for bridging research and policy (e.g., Family Impact Seminars; Pew Results First, [14, 22]). In contrast to dissemination efforts involving a one-sided flow of information to policymakers, starting with policymakers’ interest areas allows the research translation effort to be targeted in responding with evidence related to current policy windows (i.e., discrete opportunities for policy change [33, 39].

An emerging field studying URE sheds light on *why* policymakers URE and emphasizes both access to relevant research and the building of trusting interpersonal connections with scientists [21]. Some have deemed dissemination efforts focused on access to be a “first-generation” approach, which, typically share research products not intentionally chosen for their policy relevance. In contrast, “second-generation” approaches involve facilitating researcher-policymaker relationships [21]; these more potent, interactive partnership approaches can be cost-prohibitive because of the staff time required to develop relationships. Fortunately, these approaches are not necessarily mutually exclusive; insights gleaned from interacting with policymakers (via second-generation approaches) may inform and strengthen the disseminations of *relevant* research evidence, extending the reach and impact of the interactive models. As conceptualized here, an enhanced dissemination (described below) addresses critical concerns about standard dissemination practice by providing relevant and timely research evidence, which is informed by researcher-policymaker interactions, and ultimately increases opportunities for further researcher-policymaker interactions, which then inform content development for future dissemination.

### SciComm Optimizer for Policy Engagement (SCOPE) intervention

The SciComm Optimizer for Policy Engagement (SCOPE) is a replicable model for disseminating and improving the reach of research among policymakers. The following core principles of this enhanced dissemination model are intended to address common flaws critiqued in URE literature on dissemination methods [21, 23] by drawing upon theories and prior research on the best practices for bridging research and policy.

*Timely and relevant*: Policymakers’ perception of relevance may be improved by recognizing their needs and priorities, cueing individual or local relevance, responding with corresponding information, and messaging accordingly [24, 37, 40]. “Feedback loops” can be created by drawing on interactive forms of research dissemination (e.g., partnerships, conversations) to inform relevant content for broad-based dissemination efforts. In particular, this work builds from an experimentally tested model for supporting policymakers’ URE known as the Research-to-Policy Collaboration (RPC [15–17]) which facilitates nonpartisan responses to policymakers’ evidence needs by assessing their policy goals before matching them with corresponding researchers. When multiple policy staff have questions about a similar topic, that topic is determined to be politically timely and associated resources are apt for dissemination. We sought to supplement the impact of this interactive brokerage model by expanding the evidence syntheses resulting from researcher-policymaker collaborations. Researchers participating in the RPC rapid response network directly contributed to the written products that were disseminated; thus, SCOPE provided a platform for researchers to communicate directly with policymakers about issues deemed timely by partnering legislative staff. Legislative staff often identified priority areas that paralleled their pre-pandemic interests, but sought to further understand the implications of the pandemic (e.g., on overdose, violence, human trafficking, child abuse).

*Researcher-policymaker interactions*: SCOPE directly connects researchers and policymakers via email. Researchers develop fact sheets and email them to legislative officials on behalf of authors (i.e., sender is the researcher’s name and message body is a plain text, polite message). This allows the legislative officials to reply directly to the author, who was offered technical assistance (e.g., how to avoid partisan language) and logistic support (e.g., scheduling meetings). Each dissemination prompted, on average, two meaningful interactions (e.g., request to meet; answer questions; present or even testify at a hearing) between the author and a policymaker or staff. Researcher-policymaker interactions informed future disseminations in a feedback loop, which meant that the intervention was not merely a one-way “push” of information, and instead was both responsive to and facilitated interactions between researchers and policymakers.

*Brief, skimmable formats*: Policymakers have competing priorities and are constantly inundated with a barrage of information ranging across policy issue areas. This results in information overload [41]. In fact, most state policymakers have previously reported they are less likely to read “full reports” than skim information [42]. Moreover, content of research materials should be written in accessible, jargon-free, plain language [37, 43, 44]. Additionally, briefs should be succinct and focus on a singular key point that is immediately conveyed to the reader [32, 44]. Following these guidelines, research-based materials were provided in a brief format preferred by policymakers, such as 1–2 page fact sheets, briefs, or notes. These synthesized implications were based on a body of research instead of single studies and often provided examples of relevant practice.

*Continuous quality improvement (CQI)* involves cycles of planning, testing, and refining practice strategies to improve practices over time [45]. SCOPE uses a rapid-cycle evaluation method that deploys A/B field experiments to learn what approach to science communication increased the reach and visibility of disseminated fact sheets (e.g., [15, 30]). These CQI efforts serve to improve the reach of disseminations over time by informing an evolution in science communication practice.

### Study aims

**Fig. 1:**
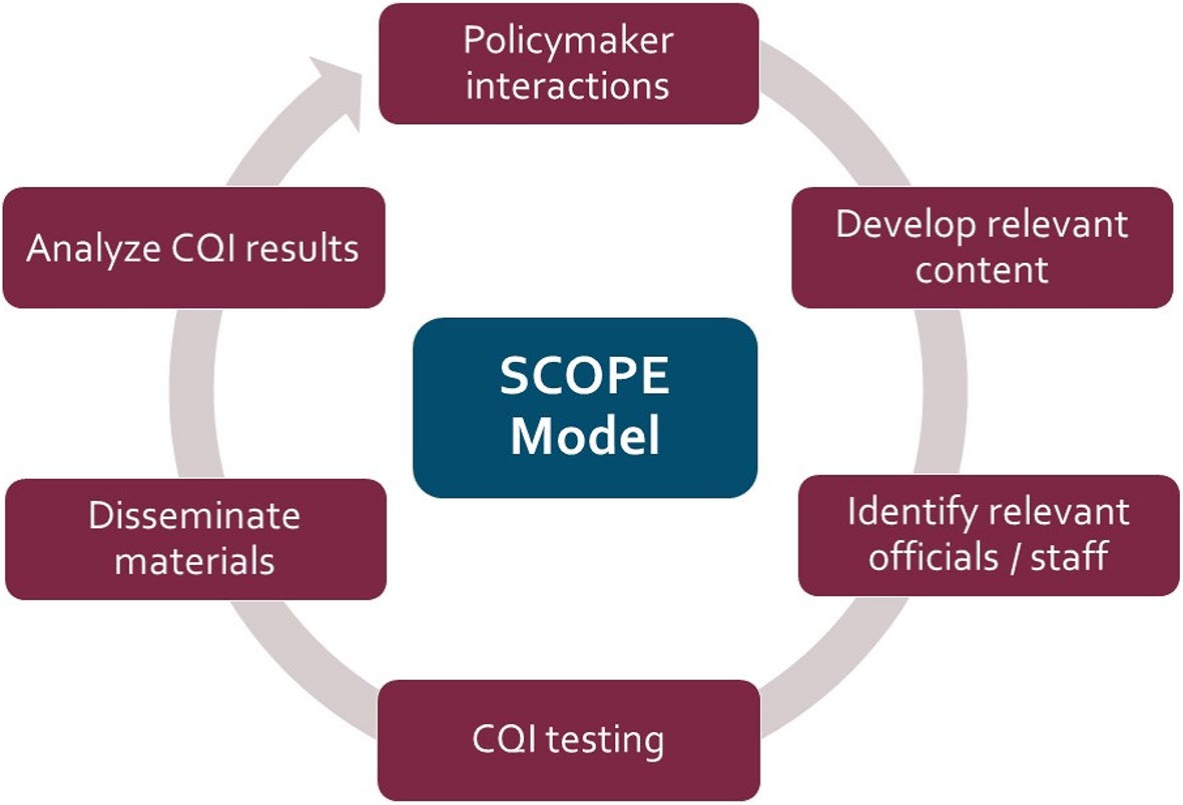
Science Communication Optimizer for Policy Engagement (SCOPE) model steps. The SCOPE model builds off of interactive models of research translation to ensure the content disseminated is relevant and timely for officials. Legislative officials who sit on relevant committees receive research syntheses directly from researchers. The content is disseminated through a platform that allows for continuous quality improvement (CQI) via field experiments that inform how to increase the reach of content over time. Thus, legislative recipients are randomized for message testing prior to dissemination and findings from those analyses inform future dissemination efforts. Direct contact between researchers and legislators fosters additional interactions that create a feedback loop in this process, leading to the development of additional content relevant to current policy priority areas

To experimentally evaluate the SCOPE intervention, a randomized controlled trial was undertaken with legislative offices (Fig. 1). This experiment sought to evaluate the effect of researcher-policymaker engagement on state legislators’ research use in social media posts during the pandemic. We hypothesized that legislators randomly assigned to receive the SCOPE intervention would be more likely to use evidence language in their public discourse related to the pandemic. Investigators felt that a study investigating ways to increase public officials’ use of scientific information in their constituent-facing communications was particularly timely in light of rampant misinformation that became prevalent in public discourse during the pandemic.

## Methods

### Intervention delivery

To experimentally evaluate the SCOPE intervention, a randomized controlled trial was undertaken with state legislative offices (Fig. 1). *Quorum* (quorum.us), a public affairs database with a client-relationship manager, was used to email selected state legislative offices and collect and manage the outcome data. Recipients in the intervention group (state legislators and their staffers, if any) were sent up to 75 emails, roughly one or two per week, containing accessible summaries of research evidence between March 1, 2020, and March 31, 2021. Evidence syntheses were relevant to a range of social issues that were affected by or salient during the pandemic (e.g., substance use, violence, workforce) in addition to public health information (e.g., mask wearing). Corresponding with substantive evidence regarding how policymakers use research, the resources disseminated were timely and relevant and brief and promoted interactions with researchers [25, 38, 45]. Recipients had the option to be removed from receiving future emails. The email body was often brief with four main parts: an introduction of the author/sender, a note on why the resource was relevant at that time, the link, and an offer to answer any questions (samples provided in Supplemental Materials). Emails were sent during normal business hours Monday through Friday. The author/sender interacted genuinely with any responses received, such as answering questions asked via email and participating in meetings. They were provided logistical support for scheduling meetings, a trained facilitator for such meetings, and suggestions on responses to questions if requested. The control group did not receive emails or any resulting opportunity to engage directly with the author/sender.

### Sample

US state and territory legislators were chosen based on committee assignments. All US states and territories were included. Specifically, US state legislators who sat on committees related to health were selected for the sample. Randomization was conducted using a simple randomization procedure in Excel by a study team member to sort 25% into the control group and 75% into the intervention. These state legislative offices were randomized such that three-quarters received the intervention (*n* = 3034) to enhance the overall availability of critical information to decision-makers during the worldwide crisis (control group *n* = 1016; see Supplemental Fig. 1 CONSORT diagram). Given the magnitude of the crisis, it seemed unethical to withhold information from more legislators than estimated for statistical power purposes. As state legislators vary in individual capacity to check emails [46, 47], the staffers of legislators in the intervention group were also included as recipients. Sample demographic data are provided in Supplemental Table 1. All present data are observational in nature; recipients were not contacted for data collection purposes for the present study.

### Measures

The dependent variable of this study is URE in COVID-19-related social media posts as indicated by Boolean phrases detecting linguistic markers for both URE and the subject matter (Supplemental Table 2). These were developed a priori based on a validated coding scheme developed in prior work [39]. Original posts (i.e., no retweets) on legislators’ official Facebook, Twitter, Medium, and YouTube accounts were searched.

*URE*: In previous work, linguistic markers have shown both to correlate with in-depth, validated coding protocols as well as demonstrate sensitivity and predictive validity for detecting change in policymakers’ URE over time [25, 39]. The study team drew from keyword phrases used in prior studies (e.g., *data shows*, *evidence-based*, *empirical research*, *representative sample*, *conduct a study* [20, 21, 41]). This initial list was revised by interviewing coders trained and experienced on a validated, in-depth, deductive coding protocol that was used to quantify URE in hundreds of bills. Coders indicated which keywords had appeared in actual legislative products to denote URE. Additionally, coders were asked *how*, rather than just *if*, these keywords are used in bills to create conceptually meaningful subdomains of URE. For example, research can be used to denote the prevalence of a problem (e.g., *studies show that 1 in 4 children*) or as part of a solution (e.g., *develop and implement an evidence-based program*), or to hold accountable the relevant parties (e.g., *assess the impact of the different programs and produce a report for Congress*), among other goals. While all those categories represent more overt expressions of research results or processes, we also captured indirect references to research through a “conceptual” research use category. Research can be used conceptually by alluding to research-based concepts related to health, such as disparities, risk factors, and social determinants, as described in disseminated content.

Throughout this process, each keyword phrase and group were reviewed to assess the prevalence of false positives (e.g., “evidence” referring to forensic evidence required more complex search phrases to assess “research evidence” or “scientific evidence”). Ultimately, eight keyword categories were created and comprised of approximately 200 research phrases. The phrases allowed for examination of a larger amount of data than what could be coded manually, given the large present sample.

*Subject matter*: Three phrases indicating relevance to the COVID-19 pandemic were identified by the study team a priori to data collection and analysis.

*Professionalism*: The Squire Index [49] was used to quantify the degree of professionalism of the members’ state legislature. The Squire Index assesses state legislatures’ capacity to create and use information for their policymaking and actively participate in policymaking. It compiles data such as number of staff, legislator salaries, and number of days in session.

### Data collection

The number of social media posts containing both subject matter and URE markers posted between March 1, 2020, and June 30, 2021 (to align with the beginning of the intervention and the end of many state legislative sessions, respectively), was used as the dependent variable for analyses. Each URE category phrase was searched within COVID-related social media posts. The counts of how many social media posts a state legislator produced that satisfied these Boolean phrases were returned using Quorum. Re-posts and shared posts (e.g., retweets) were excluded. The counts across URE categories were added to obtain a total count of social media posts related to COVID using URE. Additional data were collected during a baseline period (March 1, 2019–February 29, 2020) to test the equivalence of groups a priori by assessing the number of health-related social media posts made by state legislators in our sample.

### Analyses

A zero-inflated negative binomial regression for count data was conducted to investigate differences between the control and intervention groups’ URE within COVID-related social media posts from March 1, 2020, to June 30, 2021. The distribution of relevant social media posts was skewed positive and met the assumption of overdispersion for negative binomial regressions. Subsequent to detecting a main effect, similar secondary analyses were conducted to examine the ways that legislators used research language in social media posts. Given the distribution pattern of the outcome variable, a low interclass correlation between legislators within states, and optimal model fit indices across potential modeling options, a zero-inflated negative binomial model was selected. The rate of having no social media posts containing both URE and COVID-19 markers in this sample was 69.95%. Excessive zeros contributing to overdispersion of the data may indicate that a portion of state legislators’ social media posts were not captured or that the state legislators do not post to a public social media account. Therefore, we adjusted for the zero-inflation using two indicators that would be likely to predict whether a state legislator created a social media post with URE at all or not—the Squire Rank Index and the intervention. The Squire Index, a measure of state legislative professionalism, indicates the institutional capacity of legislatures for engagement. The Rank version orders the states based on their raw index score [49]. This measure is related to legislative capacity for creating new bills [50]. We hypothesized that capacity for engagement may influence social media activity levels, with members of more professional state legislatures being more likely to be expected to post on social media and have sufficient professional resources to be able to do so. For example, legislators in less professional legislatures are expected to hold jobs outside of their part-time legislative work and therefore may not have personal or staff time to post to social media. In contrast, legislators of more professional legislatures work full-time as a policymaker and therefore have the capacity to post to social media. Investigators accounted for the intervention in both the inflation and count parts of the model because we expected that the intervention could affect both whether someone posts URE at all as well as how often they do so.

An additional set of analyses were conducted comparing health-related social media posts with URE markers during the baseline time period in order to examine differences between groups prior to the intervention.

## Results

**Fig. 2:**
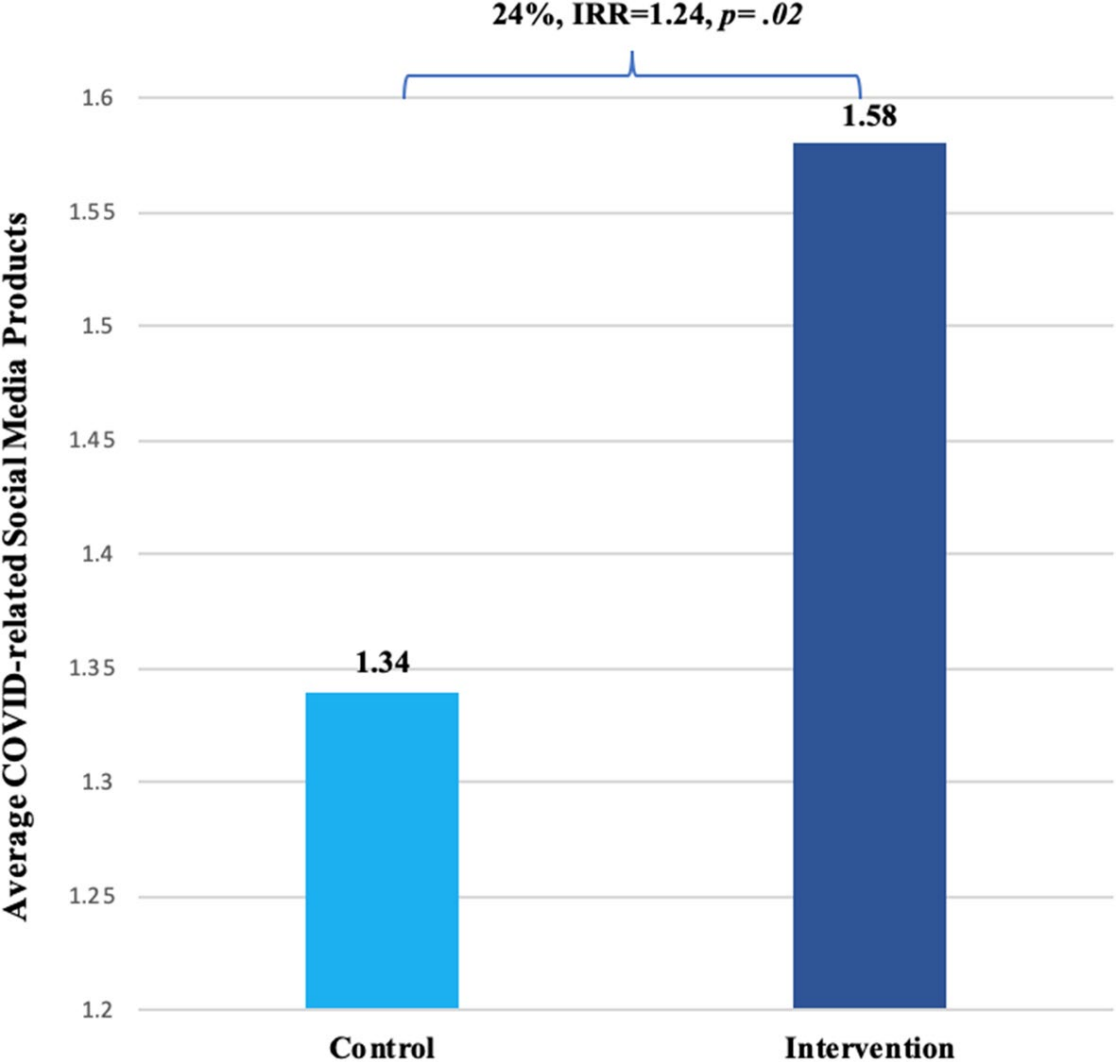
Main effect of intervention on URE in social media. State legislators receiving the intervention from the RPC produced 24% more COVID-related social media posts with URE than those not in the intervention group, controlling for the effect of state professionalism on zero-inflation

Descriptive statistics are available in Table 1. No significant group differences were found in the number of health-related social media posts using research language during the baseline time period. Results of the zero-inflated negative binomial regressions examining social media posts during the intervention period are presented in Table 2. Results showed that state legislative offices receiving the enhanced dissemination emails posted 24% more social media messages containing evidence language compared to control offices (*IRR* = 1.24, *p* = 0.02; Fig. 2). This effect size translates to an additional estimated 789 posts that used research evidence language across the sample attributable to the SCOPE intervention.

**Table 1:** Descriptive statistics

	**Control**	**Intervention**
*N* valid	998	2975
Party		
*Republican*	545 (54.6%)	1576 (53.0%)
*Democrat*	448 (44.9%)	1376 (46.2%)
*Other*	5 (0.5%)	23 (0.8%)
Chamber		
*House*	595 (59.6%)	1797 (60.4%)
*Senate*	329 (33.0%)	964 (32.4%)
*Other*	74 (7.4%)	214 (7.2%)
Gender		
*Female*	341 (34.2%)	988 (33.2%)
*Male*	657 (65.8%)	1987 (66.8%)
N terms (M (SD) (N valid))	3.67 (2.88) (979)	3.80 (3.05) (2947)
Squire rank (M (SD))	23.68 (14.72)	24.32 (14.90)
Squire score (M (SD))	0.241 (0.115)	0.238 (0.115)
URE outcomes (M (SD))		
Overall	1.36 (3.73)	1.59 (4.72)
Problem definition	0.20 (0.96)	0.21 (1.02)
Problem solution	0.06 (0.32)	0.07 (0.39)
Knowledge generation	0.08 (0.64)	0.05 (0.29)
Methods	0.07 (0.59)	0.06 (0.34)
Type of study	0.01 (0.11)	0.01 (0.09)
Data/analysis	0.10 (0.58)	0.17 (1.37)
Conceptual	0.91 (2.49)	1.12 (3.49)

**Table 2:** Intervention effect modeled with zero-inflated negative binomial regressions

	Estimate (95% CI) *p-*value									
	IRR (count model)			OR (logistic/inflation model)						
Predictor	Intervention			Intervention				State professionalism (Squire Index)		
**Main effect** (overall URE)	1.24	(1.04, 1.48)	**0.02**	1.15	(0.79, 1.68)	0.46	1.09		(1.08, 1.11)	**0.00**
**Post hoc analyses**										
Accountability	17.03	(1.78, 162.82)	**0.02**	122.73	(1.98, 7631.20)	**0.02**	1.07		(1.03, 1.11)	**0.00**
Conceptual	1.28	(1.05, 1.56)	**0.01**	1.09	(0.74, 1.60)	0.68	1.09		(1.08, 1.11)	**0.00**
Data/analytics	1.67	(1.03, 2.07)	**0.04**	0.79	(0.23, 2.43)	0.64	1.11		(1.05, 1.18)	**0.00**
Knowledge generation	0.31	(0.15, 0.64)	**0.002**	0.32	(0.12, 0.92)	**0.04**	1.04		(1.01, 1.08)	**0.01**
Methods	0.56	(0.26, 1.19)	0.13	0.53	(0.21, 1.32)	0.17	1.04		(1.01, 1.07)	**0.01**
Problem definition	1.24	(0.09, 1.74)	0.20	1.70	(0.64, 4.51)	0.28	1.08		(1.04, 1.12)	**0.00**
Problem solution	1.22	(0.60, 2.47)	0.59	1.00	(0.38, 2.66)	1.00	1.04		(1.02, 1.06)	**0.00**
Type of study	11.37	(1.70, 76.08)	**0.01**	78.26	(0.57, 13,083.41)	0.10	1.04		(1.01, 1.08)	**0.02**

This is a zero-inflated model, such that the model simultaneously conducts a logistic regression to determine predictors of whether the legislator created any posts with URE (0 vs 1 + ; i.e., the zero inflation), and a negative binomial regression to determine predictors of how frequently the legislator created posts with URE among those with more than 0 posts (the count model). We modeled the inflation with the index of state professionalism and their experimental condition (Intervention), and the count with just the experimental condition. The columns under “Logistic/inflation model” report the OR (odds ratio) and associated numbers for those predictors, and the columns under “Count model” report the IRR (incident rate ratio) for the intervention indicator

Overall URE is the count of social media posts that had any of the following eight URE type categories that follow. Control group was the reference in these analyses

The Squire Index was included in every model conducted and was a significant predictor of zero inflation for all models (*p* < 0.001). This indicates that a state’s professionalism is associated with inflated zeros such that legislators in states with low professionalism are more likely to have had zero social media posts related to health that included URE.

**Fig. 3:**
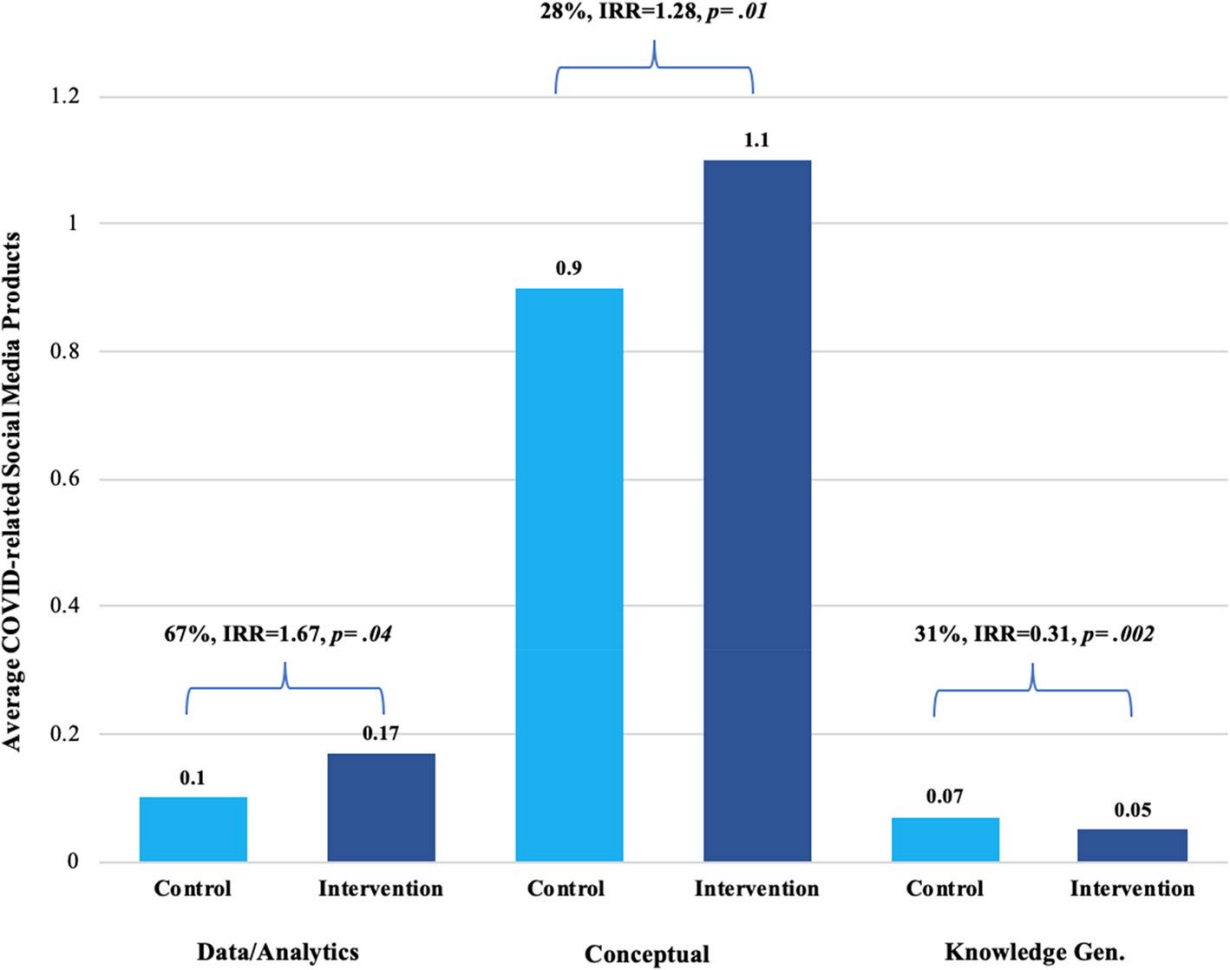
Secondary analyses: intervention effect on URE categories in social media. The relationship between the intervention and URE in social media products was driven primarily by the Data/analytics and Conceptual sub-categories. State legislators in the intervention group produced 67% more COVID-related social media posts with URE falling in the Data/analytics category than those not receiving the intervention, controlling for the effect of state professionalism on inflation. Legislators in the intervention group produced 28% more COVID-related social media posts with URE in the Conceptual category than those not receiving the intervention, controlling for the effect of state professionalism on inflation. Legislators in the control group produced 69% more social media posts with URE in the Knowledge Generation category

Secondary analyses (Fig. 3) were conducted to examine which keyword categories (i.e., problem definition, etc.) were driving this relationship, while controlling for the effect of state professionalism on zero inflation. Legislators who received the intervention produced 67% more COVID-related social media posts containing data/analytics research language than legislators in the control group (*IRR* = 1.67, *p* = 0.004), and 28% more posts with conceptual research use (*IRR* = 1.28, *p* = 0.01). Additionally, legislators in the control group produced 69% more posts with research use related to knowledge generation (*IRR* = 0.31, *p* = 0.002) and were 68% more likely to post research use related to knowledge generation at all (*OR* = 0.32,* p* = 0.04) than those in the intervention group. Analyses for Accountability and Type of Study categories, which were created prior to data collection and not otherwise examined until analyses, yielded large confidence intervals attributable to a very low, restricted range of use of these indicators; therefore, investigators did not interpret these coefficients. There were no significant differences between groups for other keyword categories (problem definition, problem solution, methods).

## Discussion

This study suggests that it is possible for theory-based, enhanced research dissemination efforts to influence state legislators’ public discourse by increasing their access to scientific evidence. This is noteworthy because science-based discourse may counteract misinformation and support the government’s role in sharing accurate health information with the public [4]. In particular, SCOPE was most effective for improving conceptual URE via reference to theoretical and research-based language, as well as data and analytic language that may reflect a type of process URE. Intervention legislators may have been more likely to emphasize high-quality methods indicating credibility behind scientific concepts. These findings correspond with research suggesting that policymakers often use research indirectly for understanding how to conceptualize problems [31], demonstrating credibility among colleagues, and educating constituents [38].

In contrast, intervention legislators posted less often about creating and disseminating evidence (i.e., knowledge generation). Perhaps intervention offices perceived existing research to be sufficient in supply, in line with the availability heuristic, therefore feeling less need for additional studies [51, 52]. Alternatively, control group legislators may have sought more research to clarify contradictory sources of information. Due to the novelty of this area of research, comparable literature to assist in this interpretation is limited. Further studies are needed to understand the relationship between exposure to research evidence and demand for additional scientific knowledge.

This work builds on literature related to research dissemination and use by addressing some consistent methodological limitations. Previous work on supporting policymakers’ access to and use of scientific information is primarily retrospective, correlational, representative of only a small portion of legislators, and subject to social desirability (i.e., rely on self-report [45]). Very little rigorous research has been done to evaluate research translation strategies [6], and this study demonstrates a feasible method of experimentation of current practices by drawing upon observational measures of impact such as public discourse. Such evaluation approaches could be highly beneficial for well-established and well-respected research synthesis centers (e.g., McMaster Health Forum, Scholar Strategy Network, and synthesis centers [53]). Not only did investigators prospectively examine the impact of a research dissemination model with experimental design, but also this study incorporated leading theories from this past research directly into the design of the present intervention. Thus, this rigorous evaluation provides some causal evidence of effectiveness for theory-based research dissemination practices that have the potential to influence legislative discourse at a time of worldwide crisis.

### Limitations and future directions

While this study revealed the potential for SCOPE to significantly impact research language use, given the nascency of this topic and methodology, there are potential limitations that warrant further study. First, the present work compared SCOPE to a control that did not receive evidence in a way we can track; there is more work to be done to compare this theory-based model to one that follows the first-generation “push” approach to dissemination.

Further, the estimates of the effect may be an underestimate, given that legislators within a state legislature are inherently connected—sharing buildings, committees, and often even staffers—which may contribute to a spillover effect. The social use of evidence for earning trust, demonstrating credibility, educating others, and enhancing debate and collaboration between lawmakers has been documented in previous studies [18]. Therefore, the control group may have been exposed to the intervention vicariously through the intervention group. The effects may be underestimated further given the social media data were restricted to posts specific to the pandemic (as opposed to broad social issues mentioned in briefs); this provided a feasible amount of data for processing capacity of the data collection platform. Additionally, due to large sample sizes involving copious amounts of analyzed text, assessing the nature of the content or veracity of research used in social media posts was beyond the scope of this study. It was not possible to do in-depth coding regarding the nature of how evidence was used in posts; thus, the current study examined overt indicators of evidence language. Future work may seek to incorporate measures of content accuracy and types of research use in order to obtain a more comprehensive understanding of policymakers’ use of high-quality scientific evidence.

Finally, it is unclear how the current pandemic context affected demand, access, and uptake of research information; therefore, the findings are not generalizable to other time periods or other topic areas (e.g., climate change) and there is a need to replicate this experiment. Converse to generalizability across issue areas is a need to differentiate strategies for different types of policymakers. For instance, while state legislators are critical for public communication and enacting new policies, agency officials may be another audience of interest for future intervention testing given their role in implementing policy. Similarly, research use patterns may vary by political affiliation as prior studies have illustrated different research use patterns, emphases, and impact of research communication approaches across party lines [16, 17, 29]. Future studies should consider experimentally testing strategies tailored to sociopolitical preferences and testing interventions by stratifying samples by political party.

SCOPE could be replicated in future science translation efforts that embed both the relational nature of the enhanced dissemination method and routinized evaluation for continuous quality improvement. To improve access to trusted, nonpartisan research evidence and to facilitate its use, research translation teams must embrace best practices such as personalizing to the needs of policymakers. Those seeking to apply these findings should adhere to the relational aspect of this work, such that the science that is distributed is drawn from interacting with policymakers, and the dissemination effort itself encourages further interactions between researchers and policymakers. More broadly, these findings could be built upon by further increasing the reach of the research syntheses in this effort, such as using different interpersonal tactics or framing strategies [28, 55, 56]. Moreover, expanding the interactive nature of this work with further optimization may have the potential to yield additional or more complex forms of URE.

## Conclusion

It is critical to the widespread implementation of evidence-based programs to promote access and use of research evidence among the people who determine the nation’s priorities, write our policies, and deem what gets funded. This work highlights the importance of developing evidence-based strategies to support decision-makers’ research use. Effective research translation approaches have the potential to improve the societal impact of scientific knowledge ranging from battle-tested practices to revolutionary studies as seen in the pandemic. As the COVID-19 pandemic has shown, the spread of both scientific information and inaccurate misinformation is complex and dynamic, which requires multifaceted research dissemination strategies. Rigorous evaluations of research translation efforts like this one are key to developing and identifying evidence-based strategies for improving the use of science.

## Supplementary Information

**Additional file 1: Supplemental Figure 1. **CONSORT Diagram. **Supplemental Table 1. **Legislative Demographic Data. **Supplemental Table 2. ** Linguistic Markers via Boolean Search Phrases for URE Categories and Subject Matter.

## Data Availability

The datasets generated and/or analyzed during the current study are available in the OSF repository, https://osf.io/f3zp9/?view_only=7b74aeb81eb84aefaa1031e318a86a30.

## Abbreviations

ICC: Intraclass correlation coefficient
SCOPE: SciComm Optimizer for Policy Engagement
URE: Use of research evidence

## References

[1] Chen E, Chang H, Rao A, Lerman K, Cowan G, Ferrara E. COVID-19 misinformation and the 2020 US presidential election. Harv Kennedy Sch Misinformation Rev. 2021;

[2] Agley J (2020). Assessing changes in US public trust in science amid the COVID-19 pandemic. Public Health.

[3] Tseng V. Transforming evidence for policy in the wake of COVID-19 [Internet]. Transforming Evidence. 2020 [cited 2020 May 12]. Available from: https://transformure.wordpress.com/2020/04/24/transforming-evidence-for-policy-in-the-wake-of-covid-19/.

[4] Tsao SF, Chen H, Tisseverasinghe T, Yang Y, Li L, Butt ZA (2021). What social media told us in the time of COVID-19: a scoping review. Lancet Digit Health.

[5] Bunnell R, Ryan J, Kent C, Committee CO of S and CE in S (2021). Toward a new strategic public health science for policy, practice, impact, and health equity. Am J Public Health.

[6] Oliver K, Hopkins A, Boaz A, Guillot-Wright S, Cairney P (2022). What works to promote research-policy engagement?. Evid Policy.

[7] Funk C. Mixed Messages about Public Trust in Science - ProQuest [Internet]. 2017 [cited 2021 Dec 13]. Available from: https://www.proquest.com/docview/2177530522/fulltextPDF/19BD756F6BD2475EPQ/1?accountid=10226.

[8] Blake A. Americans’ increasing distrust of science — and not just on climate change. Washington Post [Internet]. 2015 [cited 2021 Dec 13]; Available from: https://www.washingtonpost.com/news/the-fix/wp/2015/01/30/americans-increasing-distrust-of-science-and-not-just-on-climate-change/.

[9] Beck J. The Challenge of Fighting Mistrust in Science [Internet]. The Atlantic. 2017 [cited 2021 Dec 13]. Available from: https://www.theatlantic.com/science/archive/2017/06/the-challenge-of-fighting-mistrust-in-science/531531/.

[10] Funk C. Key findings about Americans’ confidence in science and their views on scientists’ role in society [Internet]. Pew Research Center. 2020 [cited 2021 Dec 10]. Available from: https://www.pewresearch.org/fact-tank/2020/02/12/key-findings-about-americans-confidence-in-science-and-their-views-on-scientists-role-in-society/.

[11] Kreps SE, Kriner DL (2020). Model uncertainty, political contestation, and public trust in science: evidence from the COVID-19 pandemic. Sci Adv.

[12] Bogenschneider K, Little OM, Johnson K (2013). Policymakers’ use of social science research: looking within and across policy actors. J Marriage Fam.

[13] Kennedy B, Tyson A, Funk C. Americans’ Trust in Scientists, Other Groups Declines. Pew Research Center Science & Society. 2022 [cited 2022 Jul 25]. Available from: https://www.pewresearch.org/science/2022/02/15/americans-trust-in-scientists-other-groups-declines/.

[14] Gollust SE, Seymour JW, Pany MJ, Goss A, Meisel ZF, Grande D (2017). Mutual distrust: perspectives from researchers and policy makers on the research to policy gap in 2013 and recommendations for the future. Inq J Health Care Organ Provis Financ.

[15] Stokes DC, Purtle J, Meisel ZF, Agarwal AK (2021). State legislators’ divergent social media response to the opioid epidemic from 2014 to 2019: longitudinal topic modeling analysis. J Gen Intern Med.

[16] Guntuku SC, Purtle J, Meisel ZF, Merchant RM, Agarwal A (2021). Partisan differences in Twitter language among US legislators during the COVID-19 pandemic: Cross-sectional Study. J Med Internet Res.

[17] Engel-Rebitzer E, Stokes DC, Meisel ZF, Purtle J, Doyle R, Buttenheim AM (2022). Partisan differences in legislators’ discussion of vaccination on Twitter during the COVID-19 era: natural language processing analysis. JMIR Infodemiology.

[18] Bogenschneider K, Day E, Parrott E (2019). Revisiting theory on research use: turning to policymakers for fresh insights. Am Psychol.

[19] Payán DD, Lewis LB (2019). Use of research evidence in state health policymaking: menu labeling policy in California. Prev Med Rep.

[20] Scott J, Lubienski C, DeBray E, Jabbar H. The intermediary function in evidence production, promotion, and utilization: the case of educational incentives. In: Using research evidence in education. Springer; 2014. p. 69–89.

[21] Boaz A, Davies H. What works now?: evidence-informed policy and practice. Policy Press; 2019.

[22] Bogenschneider K, Day E, Bogenschneider BN (2022). When policymakers are asked: why and how polarization varies across states. Polit Res Q.

[23] Tseng V (2012). The uses of research in policy and practice.

[24] Scott J, Pugel J, Fernandes M, Cruz K, Long E, Giray C, et al. Cutting through the noise during crisis by enhancing the relevance of research to policymakers. Evid Policy J Res Debate Pract [Internet]. 2022 [cited 2022 May 13]; Available from: https://bristoluniversitypressdigital.com/view/journals/evp/aop/article-10.1332-174426421X16535828173307/article-10.1332-174426421X16535828173307.xml. 10.1332/174426421x16535828173307 PMC12060730 40343080

[25] Crowley DM, Scott JT, Long EC, Green L, Israel A, Supplee L, et al. Lawmakers’ use of scientific evidence can be improved. Proc Natl Acad Sci [Internet]. 2021 Mar 2 [cited 2021 Feb 16];118(9). Available from: https://www.pnas.org/content/118/9/e2012955118. 10.1073/pnas.2012955118 PMC7936366 33593938

[26] Scott JT, Ingram AM, Nemer SM, Crowley DM (2019). Evidence-based human trafficking policy: opportunities to invest in trauma-informed strategies. Am J Community Psychol.

[27] Yanovitzky I, Weber M (2020). Analysing use of evidence in public policymaking processes: a theory-grounded content analysis methodology. Evid Policy J Res Debate Pract.

[28] Long EC, Pugel J, Scott JT, Charlot N, Giray C, Fernandes MA (2021). Rapid-cycle experimentation with state and federal policymakers for optimizing the reach of racial equity research. Am J Public Health.

[29] Purtle J, Nelson KL, Gebrekristos L, Lê-Scherban F, Gollust SE (2022). Partisan differences in the effects of economic evidence and local data on legislator engagement with dissemination materials about behavioral health: a dissemination trial. Implement Sci.

[30] Nutley SM, Walter I, Davies HT. Using evidence: How research can inform public services. Policy press; 2007.

[31] Weiss CH (1979). The many meanings of research utilization. Public Adm Rev.

[32] Brownson RC, Eyler AA, Harris JK, Moore JB, Tabak RG (2018). Research full report: getting the word out: new approaches for disseminating public health science. J Public Health Manag Pract.

[33] Zane SN, Welsh BC (2018). Toward an “age of imposed use”? Evidence-based crime policy in a law and social science context. Crim Justice Policy Rev.

[34] Scott JT, Prendergast S, Demeusy E, McGuire K, D. Max Crowley. Trends and opportunities for bridging prevention science and US federal policy. Prev Sci. 2022;1–10. 10.1007/s11121-022-01403-2 PMC12001977 35930099

[35] Kingdon JW (1984). Agendas, alternatives, and public policies.

[36] Mackie TI, Sheldrick RC, Hyde J, Leslie LK (2015). Exploring the integration of systems and social sciences to study evidence use among child welfare policy-makers. Child Welfare.

[37] Ashcraft LE, Quinn DA, Brownson RC (2020). Strategies for effective dissemination of research to United States policymakers: a systematic review. Implement Sci.

[38] Bogenschneider K, Corbett T. Evidence-based policymaking: envisioning a new era of theory, research, and practice. Routledge; 2021. 464 p.

[39] Diaz, B. A., Pugel, J., Phutane, A., Zhang, L., Green, L., Hoffman, J., et al. Intra-stage mixed methods as strategies for meta-inferences: a reflexive report on the use of research evidence in federal policymaking. J Mix Methods Res. Under review; 10.1016/j.evalprogplan.2024.102469 PMC11907767 39047657

[40] Macoubrie J, Harrison C. Human services research dissemination: what works? Office of Planning, Research and Evaluation, US Administration for Children and Families; 2013.

[41] Brownson RC, Royer C, Ewing R, McBride TD (2006). Researchers and policymakers. Am J Prev Med.

[42] Sorian R, Baugh T (2002). Power of information: closing the gap between research and policy. Health Aff (Millwood).

[43] Bogenschneider K (2020). Positioning universities as honest knowledge brokers: best practices for communicating research to policymakers. Fam Relat.

[44] Goldstein CM, Murray EJ, Beard J, Schnoes AM, Wang ML (2020). Science communication in the age of misinformation. Ann Behav Med.

[45] Atukpawu-Tipton G, Poes M. Rapid Cycle Evaluation at a Glance (OPRE Report #2020–152) [Internet]. Office of Planning, Research, and Evaluation, U.S. Department of Health and Human Services; 2020 [cited 2022 Apr 17]. Available from: https://www.acf.hhs.gov/opre/report/rapid-cycle-evaluation-glance.

[46] Cooper CA (2002). E-mail in the state legislature: evidence from three states. State Local Gov Rev.

[47] Pugel J, Long EC, Fernandes M, Cruz K, Giray C, Crowley DM, et al. Who is listening? Profiles of policymaker engagement with scientific communication. Policy Internet. 2021;1–16. 10.1002/poi3.273 PMC9216211 35757292

[48] Long EC, Scott JT, Craig LE, Prendergast S, Pugel J, Crowley DM. How substance use prevention research gets used in United States federal policy. Addiction. 2022; 10.1111/add.15874 PMC12005086 35293062

[49] Squire P (2017). A Squire Index Update. State Polit Policy Q.

[50] Jansa JM, Hansen ER, Gray VH (2019). Copy and paste lawmaking: legislative professionalism and policy reinvention in the states. Am Polit Res.

[51] Tversky A, Kahneman D (1974). Judgment under uncertainty: heuristics and biases. Science.

[52] Greifeneder R, Bless H, Pham MT (2011). When do people rely on affective and cognitive feelings in judgment?. A review Personal Soc Psychol Rev.

[53] Elliott H, Popay J (2000). How are policy makers using evidence? Models of research utilisation and local NHS policy making. J Epidemiol Community Health.

[54] Baron JS, Specht A, Garnier E, Bishop P, Campbell CA, Davis FW (2017). Synthesis centers as critical research infrastructure. Bioscience.

[55] Weiss CH. Research for policy’s sake: the enlightenment function of social research. Policy Anal. 1977;531–45.

[56] Fernandes M, Scott T, Long E, Pugel J, Cruz K, Giray C, et al. Rapport building communications for optimizing science dissemination. 2021 Jun.

