# Supplementary material for: SciComm Optimizer for Policy Engagement: a randomized controlled trial of the SCOPE model on state legislators’ research use in public discourse
Source: Implement Sci. 2023 May 5;18:12. doi: 10.1186/s13012-023-01268-1 (PMC10160730; doi:10.1186/s13012-023-01268-1)
Supplement: Supplementary file 1 — Additional file 1: Supplemental Figure 1. CONSORT Diagram. Supplemental Table 1. Legislative Demographic Data. Supplemental Table 2. Linguistic Markers via Boolean Search Phrases for URE Categories and Subject Matter. [file 13012_2023_1268_MOESM1_ESM.docx]

SciComm Optimizer for Policy Engagement: A Randomized Controlled Trial of the SCOPE Model on State Legislators’ Research Use in Public Discourse

**Supplementary Materials**

**Supplemental Figure 1.** CONSORT Diagram


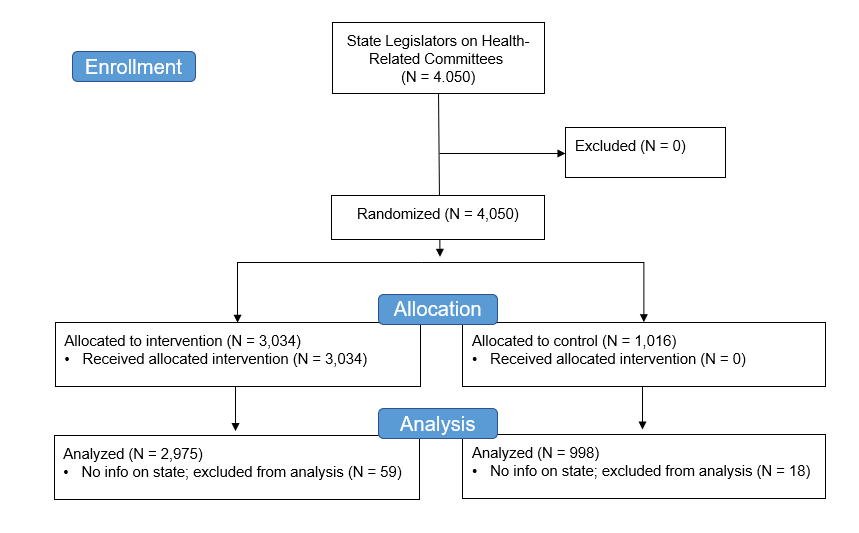


**Note:** 4,050 state legislators assigned to health-related committees were selected for participation in this study. 75% of legislators were randomized to receive the RPC intervention and 25% were randomized to the control group.  Randomization was not balanced given ethical considerations of withholding information during the crisis from more people than estimated necessary.

**Supplemental Table 1.** Legislative Demographic Data

|  | Control (*N=*1,016) | Intervention (*N*= 3,034) |
| --- | --- | --- |
| Age – M(SD) | 57.61 (13.24) | 58.98 (12.78) |
| Gender – N(%) |  |  |
| Male | 670 (65.94%) | 2,038 (67.17%) |
| Female | 346 (34.06%) | 996 (32.83%) |
| Chamber – N(%) |  |  |
| Senate | 331 (32.58%) | 989 (32.60%) |
| House | 681 (67.02%) | 2,039 (67.20%) |
| Other | 4 (0.40%) | 6 (0.20%) |
| Party – N(%) |  |  |
| Republican | 545 (53.64%) | 1,576 (51.94%) |
| Democrat | 452 (44.49%) | 1,381 (45.52%) |
| Other | 19 (1.87%) | 77 (2.54%) |
| Terms Served – M(SD) | 3.65 (2.87) | 3.78 (3.01) |
| Member Ethnicity – N(%) |  |  |
| White | 810 (79.96%) | 2,393 (79.06%) |
| Black | 109 (10.76%) | 338 (11.17%) |
| Hispanic | 65 (6.42%) | 190 (6.28%) |
| Other | 32 (2.86%) | 110 (3.50%) |

**Supplemental Table 2.** Linguistic Markers via Boolean Search Phrases for URE Categories and Subject Matter

| **Keyword Category** | **Description** | **Example Search Phrase** |
| --- | --- | --- |
| COVID-19 | Subject matter phrases related to the COVID-19 pandemic. | ("COVID-19" OR "pandemic" OR "coronavirus") |
| Accountability | Language describing the use of performance measures, data, and results were most often used for evaluating policy programs. | (“quality improvement” OR “measurable objectives” OR “impact analysis”) |
| Conceptual | Research-based concepts as derived from content provided in researchers’ fact sheets. | (“disparities” OR “risk factors” OR “social determinants” OR “trauma-informed”) |
| Data/Analytics | Technical language related to types of data and statistical analyses. | (“observational data” OR “meta-analysis” OR “statistically validate”) |
| Knowledge Generation | Language proposing to fund research on developing and disseminating evidence of best practices. | (“implement research” OR “develop studies” OR “dissemination”) |
| Methods | Technical language related to study design. | (“sample size” OR “hypotheses” OR “control group”) |
| Problem Definition | Language directly referencing existing data or studies was most often used to describe the problem, its causes, or consequences. | ("data indicate" OR "evidence indicates" OR "studies assessing" OR “existing research”) |
| Problem Solution | Language that characterized the effectiveness of strategies for addressing the problem. | (“research informed” OR “evidence based” OR “data supports” OR “data informed”) |

*Example email bodies*

Example Email 1:

Subject line:

Law enforcement and implicit bias training

Email body:

Good morning {{appropriate_greeting}},

Hope your week is going well and thank you for serving {{recipient_state_name}}! This month, I co-authored a fact sheet on how traditional implicit bias trainings do not reduce police officers' biases long-term, and what evidence-based solutions are likely more impactful. You can read it here: [https://www.research2policy.org/police-reform/implicit-bias-police-training%3A-what-works%3F](https://www.research2policy.org/police-reform/implicit-bias-police-training:-what-works)

Please let me know if you have any questions or comments about this. I would be happy to further discuss implicit bias trainings.

Warmly,

[Author’s first name]

[Author’s signature block]

Data on email open and click rates will be used for research and quality improvement. If you have questions about this research, you may contact the Office for Research Protections at (XXX) XXX-XXXX, [ORProtections@XXX.edu](mailto:ORProtections@XXX.edu), or [investigator name] at [XXX@XXX.edu](mailto:XXX@XXX.edu). If you would like to change your communication preferences, please contact [admin name] at [admin@XXX.org](mailto:admin@XXX.org).

Example Email 2:

Subject line:

Challenges in student access to technology during COVID

Email body:

Hi {{appropriate_greeting}},

Hope you’re doing well. I created a brief fact sheet about how and why youth living in poverty are less well-equipped to do virtual learning, and a series of policy options to consider as schools prepare to begin teaching again in the next month or so. I thought you may find it useful. You can view the fact sheet here:

<https://www.research2policy.org/covid19-digital-divide>

Please reach out if you have any questions about how we can support students through this pandemic.

Thanks,

[Author’s first name]

[Author’s signature block]

Data on email open and click rates will be used for research and quality improvement. If you have questions about this research, you may contact the Office for Research Protections at (XXX) XXX-XXXX, [ORProtections@XXX.edu](mailto:ORProtections@XXX.edu), or [investigator name] at [XXX@XXX.edu](mailto:XXX@XXX.edu). If you would like to change your communication preferences, please contact [admin name] at [admin@XXX.org](mailto:admin@XXX.org).
